# Cross-national variation in how members of the community define flourishing mental health

**DOI:** 10.1177/00207640251323345

**Published:** 2025-02-28

**Authors:** Richard Andrew Burns, Kerry Sargent, Dimity Ann Crisp

**Affiliations:** 1 National Centre for Epidemiology and Population Health, Australian National University, Canberra, ACT, Australia; 2 Discipline of Psychology, Faculty of Health, University of Canberra, Canberra, ACT, Australia

**Keywords:** Flourishing, mental health, psychological wellbeing, subjective wellbeing, wellbeing

## Abstract

**Background:**

The experience of flourishing (i.e. high wellbeing) is informing our understanding of psychological health beyond psychopathology.

**Aims:**

This study examines whether community members define their sense of flourishing in terms of the presence of wellbeing and/or the absence of psychopathology.

**Methods:**

Participants (*n* = 1,094) were stratified by sex and age (18–39 years, 40–59 years and 60 years+), resided in Australia, the United Kingdom, Singapore, South Africa and Malaysia. Participants were presented with 12 items from the European Social Survey Wellbeing Module and 9 symptoms from the Diagnostic Statistical Manual for Major Depressive Disorder and Generalized Anxiety Disorder; mental health items were rephrased to reflect an absence of psychopathology. Respondents selected and ranked the five statements that best reflected their sense of flourishing.

**Results:**

Wellbeing statements were the most frequently endorsed items for example, ‘Feeling calm and peaceful’, ‘Life is valuable and worthwhile’, ‘Having people who care’ and ‘Feeling positive about oneself’, but they were only endorsed by approximately 35% to 38% of respondents. Three pathology items were amongst the top 10 items endorsed.

**Conclusions:**

That not one indicator was endorsed by the majority of respondents suggests that flourishing definitions of positive mental health need to be defined by both the presence of wellbeing and absence of psychopathology. Notably, there were few between-nation differences in items endorsed, and those differences reported were not of a large magnitude suggesting consistency in the endorsement of indicators between nations.

## Introduction

The experience of wellbeing, sometimes referred to as positive mental health, contrasts with psychopathology and is increasingly informing clinical practice and mental health policy (Corbin et al., 2021; Government, 2020; Sweden, 2017; Treasury, 2019). Flourishing is a term commonly used to describe the experience of high wellbeing. Agenor et al. (2017) have described flourishing an ‘immature’ concept and this criticism can partly be attributed to Jingle-Jangle wellbeing fallacies (Marsh et al., 2020), whereby similarly named constructs actually measure different things (Jingle) or where differently named constructs actually measure similar things (Jangle). Similar and dissimilar components are purported to ‘measure’ flourishing. Depending on the framework utilized, Hone et al. (2014) demonstrated a relatively low concordance (from 24% to 47%) between four common flourishing measures in flourishing prevalence.

The paper seeks to contribute to a developing framework (Burns et al., 2022) in which community members define flourishing both in terms of wellbeing and the extent to which they are free of pathology. In a restricted-choice and ranking pseudo-experimental design, community members, defined flourishing as both the experience of wellbeing and the absence of pathology, and no single indicator was consistently endorsed by all participants as reflecting flourishing (Burns et al., 2022). This suggests that in contrast to most existing flourishing frameworks, which are defined in terms of wellbeing only (Diener et al., 2010; Hone et al., 2014; Huppert & So, 2013; Seligman, 2018), community members emphasise absence of psychopathology as well as the presence and experience of wellbeing for defining flourishing mental health. There is however a paucity of research examining potential cross-national differences in how community members define what it means to flourish. Following Burns et al (2022) and Delle Fave et al. (2011), we examine the consistency of their findings in an age-sex balanced cross-national sample of adults across five nations and propose the following aims:

Aim 1: Respondents will select a combination of wellbeing and mental health indicators as reflecting flourishing. No single indicator will be selected by a majority of respondents.
Aim 2: We expect community members from different nations to define flourishing in similar ways. There will be no substantive between-nation differences in the extent to which individual indicators are endorsed.
Aim 3: Following previous findings (Burns et al., 2022) the endorsement of individual indicators as reflecting flourishing will be unrelated to key socio-demographic factors

## Methods

### Participants

A Qualtrics online data collection service was used to randomly sample participants aged 18 years and over residing in Australia, the United Kingdom, Singapore, South Africa and Malaysia, and who were proficient in English. Respondents self-select to join Qualtrics panels and are reimbursed for participating in online surveys. Qualtrics is one of the largest online panel and survey providers and undertakes robust respondent checks to ensure quality samples that reflect the national population from which they are drawn (Belliveau et al., 2022; Boas et al., 2020). Data quality is generally higher than MTurk, Soapbox Sample, and social media sources, similar to Dynata, and either comparable or slightly lower than Prolific (Armstrong et al., 2021; Ibarra et al., 2018; Peer et al., 2022). Compared to mail and telephone surveys, online surveys are more cost-effective, and more accessible to under-represented members of the broader population, although quality of samples may vary between providers (Wright, 2017).

As wellbeing varies by age and sex, quotas were employed to generate evenly distribute sex and age-groups (18 to 39 years, 40 to 59 years and 60 years+) within nations. With five nations, 2 sex and 3 age groups, an a-priori power analysis estimated a total sample size of *n* = 1,004 assuming a small effect size, *p* = .05, and Power = 0.80. A total of 1,094 participants responded to the survey, of which a final sample of 1,044 met the eligibility and fully completed the survey. All procedures involving human subjects/patients were approved by The Australian National University Human Research Ethics Committee approved the research study (Protocol #: 2022/203). Written consent was obtained from all participants.

### Measures

#### Ranking importance of mental health and wellbeing indicators to flourishing

Participants were asked to choose five statements from a list of 21 statements that they thought best reflected flourishing in life. These statements were derived from the European Social Survey (ESS) Wellbeing module, and indicators of mental health were drawn from DSM five diagnoses for Major Depressive Disorder (MDD) and Generalized Anxiety Disorder (GAD) (American Psychiatric Association, 2013). Participants were then asked to rank their five statements in order of importance.

The 11 wellbeing statements derived from the ESS Wellbeing module included items such as ‘Feeling close to community and people in the local area’; ‘Having a lot of energy’; ‘Feeling very positive about oneself’; ‘Being optimistic about the future’; ‘Having a sense of accomplishment’; ‘Being interested in learning new things’. The nine mental health indicators drawn from DSM five MDD and GAD were rephrased with a positive valence to match the valence of the wellbeing items. For example, the criterion for the presence of depressed mood was restated as ‘Not experiencing depressed mood’, and excessive worries and anxiety was rephrased as ‘Being free of excessive worries and anxieties that are difficult to control’.

#### Covariates

Several covariates were included to examine potential moderators for which statements were endorsed by community respondents. This included prior level of contact with mental health, their own level of wellbeing and psychological distress, and key socio-demographic characteristics.

##### Level of contact with mental health

The amount of experience participants had with mental illness was measured using the 12-item Level of Contact Report (Holmes et al., 1999). The scale lists 12 situations of experiencing people with mental illness that range in familiarity from ‘I have observed in passing a person I believe may have had a mental illness’ to ‘I live with a person with mental illness’. Participants indicated the statements that were true for them, with a higher score reflecting greater exposure to people with mental illness.

##### Kessler psychological distress scale

The 10-item Kessler Psychological Distress Scale (K-10; Kessler et al., 2002) assessed participants’ current psychological health. The scale consists of non-specific symptoms of psychological disturbance experienced in the last 30 days, with scores ranging from 1 *none of the time* to 5 *all of the time*. Higher scores indicate higher psychological distress. Cronbach’s alpha for the current study was α = .95.

##### Warwick Edinburgh mental wellbeing scale

Current wellbeing was measured using the 14-item Warwick Edinburgh Mental Wellbeing Scale (WEMWBS; Stewart-Brown & Janmohamed, 2008). Participants rated their experience of stated thoughts and feelings in the last 2 weeks, from *none of the time* to 5 *all of the time*. Statements included ‘I’ve been feeling optimistic about the future’, ‘I’ve been dealing with problems well’ and ‘I’ve been interested in new things’. Higher scores reflect higher levels of wellbeing. Cronbach’s alpha for the current study was α = .96.

##### Socio-demographics

Self-reported sex (Male, Female), chronological age in years and partner status (currently partnered, not partnered), were also asked.

#### Analyses

Socio-demographic characteristics of respondents were reported overall and by nation; chi-square and one-way ANOVA identified between-nation differences in these characteristics with Tukey post-hoc analysis to compare individual nations. Proportions of the endorsement of individual items were then estimated; between-nation differences were compared using Chi-Square. Rank score of the individual indicators are reported and one-way ANOVA assessed global between-nation differences in the mean ranks with Tukey post-hoc analysis to compare individual nations. Proportions of the individual ranks were then estimated, and between-nation differences were compared using Chi-Square. We report exact probability values, but noting the overall sample size, we focus on interpreting results reported with high levels of probability (*p* < .001). We also balance statistical significance with the substantive magnitude of the effects, or otherwise, of the reported differences.

## Results

### Sample socio-demographic and health characteristics

Table 1 outlines the socio-demographic and mental health characteristics of participants by nation. The total sample is reported in Supplemental Table 1. Owing to quota-sampling, there were no between-nation differences on sex or age. The majority of respondents were partnered and there was limited evidence of a small between-nation difference in partnered status (*Χ*² (4) = 10.98; *p* = .028). Australian and United Kingdom respondents reported slightly lower proportions of ‘partnered’ status.

**Table 1.** Socio-demographic and health characteristics of the participants.

	Australia (*N* = 208)	United Kingdom (*N* = 205)	Singapore (*N* = 210)	South Africa (*N* = 209)	Malaysia (*N* = 212)	Between nation differences
	*M* (*SD*)	*M* (*SD*)	*M* (*SD*)	*M* (*SD*)	*M* (*SD*)	
Male, *N* (%)	104 (50.0%)	102 (49.8%)	103 (49.1%)	107 (51.2%)	105 (49.5%)	*Χ*² = 0.2, *p* = .995
Partnered, *N* (%)	134 (64.4%)	135 (65.9%)	159 (75.7%)	155 (74.2%)	156 (73.6%)	*Χ*² = 10.9, *p* = .028
Age	48.89 (16.98)	48.00 (16.33)	47.03 (15.91)	46.52 (16.85)	46.13 (15.36)	*F* = 1.0, *p* = .405
Level of contact	8.02 (4.39)	9.02 (3.49)	6.60 (4.05)	8.30 (3.28)	6.67 (3.72)	*F* = 16.2, *p* < .001
K-10	22.44 (10.58)	23.17 (10.23)	21.39 (9.70)	20.60 (8.12)	19.59 (7.49)	*F* = 4.91, *p* = .001
Wellbeing	45.74 (11.48)	44.84 (11.63)	46.61 (10.79)	49.61 (10.95)	49.41 (9.01)	*F* = 8.4, *p* < .001

The mean level of contact scores suggests respondents had moderate contact with people with mental illness overall. Mean K10 and wellbeing scores indicate the overall sample had moderate levels of psychological distress and wellbeing in general. Between-nation differences were found for level of contact (*F* [4, 1044] = 16.18; *p* < .001), K10 (*F* [4, 1044] = 4.18; *p* = .002) and wellbeing scores (*F* [4, 1044] = 8.08; *p* < .001). United Kingdom respondents had higher mean levels of contact compared to Singaporean (*p* < .001), and Malaysian respondents (*p* < .001), while Singaporean and Malaysian respondents had lower mean levels of contact compared to Australian (Singapore: *p* = .001; Malaysia: *p* = .002) and South African respondents (Singapore; *p* < .001; Malaysia: *p* < .001). United Kingdom respondents reported higher psychological distress than Malaysian respondents (*p* = .001), whilst Australian and the United Kingdom respondents had significantly lower mean wellbeing scores than South African (Australia: *p* = .002; UK: *p* < .001), and Malaysian respondents (Australia: *p* = .005; UK: *p* < .001). The statistical significance of these differences should be balanced by the substantive magnitude of these differences.

### Mental health and wellbeing indicators of flourishing

**Figure 1.**
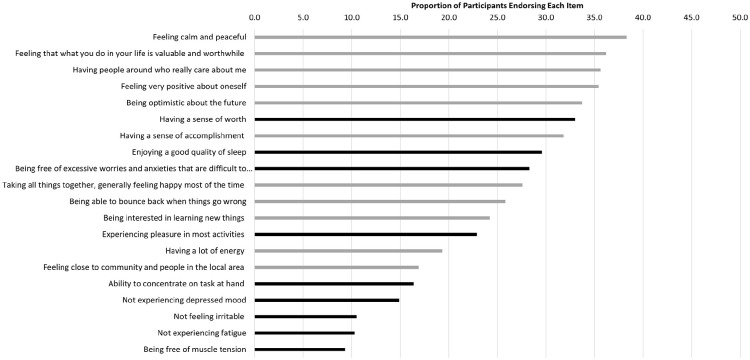
Proportion of respondents endorsing wellbeing & mental health indicators, total sample (black bars reflect mental health indicators; grey bars reflect wellbeing indicators).

The proportion of participants who endorsed each indicator is provided in Figure 1; the numerical proportion of the sample endorsing each item is reported in Supplemental Table 2. In line with our first aim, no single indicator was endorsed by a majority of respondents. Compared to the mental health indicators, the wellbeing indicators were generally more strongly endorsed as important to flourishing, specifically the wellbeing indicators ‘*Feeling calm and peaceful*’, ‘*Feeling that what you do in your life is valuable and worthwhile*’, ‘*Having people around who really care*’ and ‘*Feeling very positive about oneself*’. However, three mental health indicators (sense of worth, quality of sleep, and free of excessive worries and anxieties) were ranked in the top 10 indicators endorsed by respondents. The somatic mental health indicators were the least endorsed items (e.g. fatigue, muscle tension), and to an extent so were the absence of pathological feeling components (e.g. depressed mood, irritability, ability to concentrate).

There were very few overall differences in proportions of participants endorsing wellbeing (Table 2) or mental health (Table 3) indicators between nations. A visual comparison between nations is also available in Supplemental Figure 1. The mental health indicator ‘*Ability to concentrate on the task at hand*’ was endorsed less by South African (10.5% [*SE* = 2.1]) and Malaysian respondents (10.8% [*SE* = 2.1]) compared to respondents from the other nations (*Χ*² (4) = 17.55: *p* = .002), and *‘Not experiencing fatigue’* was endorsed less by Malaysian respondents (5.2% [*SE* = 1.5]; *Χ*² (4) = 16.47: *p* = .002). The extent of these differences should be moderated by the low prevalence by which they were endorsed by the overall sample; simply, small between-nation differences were reported on the less-endorsed indicators.

**Table 2.** Proportion of participants endorsing wellbeing indicators as flourishing, by nation.

Wellbeing Indicators	Australia	United Kingdom	Singapore	South Africa	Malaysia	Between nation differences
	% (*SE*)	% (*SE*)	% (*SE*)	% (*SE*)	% (*SE*)	
Feeling calm and peaceful	38.5 (3.4)	30.7 (3.2)	41.0 (3.4)	34.4 (3.3)	46.7 (3.4)	*Χ* ² = 13.23, *p* = .010
Feeling that what you do in your life is valuable and worthwhile	31.3 (3.2)	33.7 (3.3)	31.9 (3.2)	41.6 (3.4)	42.5 (3.4)	*Χ* ² = 10.71, *p* = .030
Having people around who really care about me	32.7 (3.3)	36.1 (3.4)	31.0 (3.2)	36.4 (3.3)	42.0 (3.4)	*Χ* ² = 6.58, *p* = .160
Feeling very positive about oneself	27.9 (3.1)	38.0 (3.4)	32.4 (3.2)	41.6 (3.4)	37.3 (3.3)	*Χ* ² = 10.46, *p* = .033
Being optimistic about the future	35.6 (3.3)	29.3 (3.2)	30.5 (3.2)	38.8 (3.4)	34.4 (3.3)	*Χ* ² = 5.55, *p* = .236
Having a sense of accomplishment	27.9 (3.1)	27.3 (3.1)	39.0 (3.4)	36.8 (3.3)	27.8 (3.1)	*Χ* ² = 12.45, *p* = .014
Taking all things together, generally feeling happy most of the time	27.4 (3.1)	26.3 (3.1)	30.5 (3.2)	24.9 (3.0)	28.8 (3.1)	*Χ* ² = 1.96, *p* = .744
Being able to bounce back when things go wrong	24.5 (3.0)	24.4 (3.0)	21.9 (2.9)	28.7 (3.1)	29.2 (3.1)	*Χ* ² = 4.30, *p* = .367
Being interested in learning new things	16.8 (2.6)	25.4 (3.0)	23.3 (2.9)	28.7 (3.1)	26.9 (3.0)	*Χ* ² = 9.54, *p* = .049
Having a lot of energy	20.7 (2.8)	22.0 (2.9)	23.8 (2.9)	12.4 (2.3)	17.4 (2.6)	*Χ* ² = 10.72, *p* = .030
Feeling close to community and people in the local area	16.3 (2.6)	17.6 (2.6)	13.8 (2.4)	19.1 (2.7)	17.5 (2.6)	*Χ* ² = 2.33, *p* = .675

**Table 3.** Proportion of participants endorsing mental health indicators as flourishing, by nation.

Mental Health Indicators	Australia	United Kingdom	Singapore	South Africa	Malaysia	Between nation differences
	% (*SE*)	% (SE)	% (SE)	% (SE)	% (SE)	
A sense of worthlessness	36.7 (3.3)	35.0 (3.3)	27.4 (3.1)	37. (3.4)2	28.6 (3.1)	*Χ* ² = 9.33, *p* = .053
Poor quality sleep	30.3 (3.2)	30.7 (3.2)	29.0 (3.1)	25.4 (3.0)	32.5 (3.2)	*Χ* ² = 2.89, *p* = .576
Excessive worries and anxieties that are difficult to control	30.3 (3.2)	26.3 (3.1)	31.4 (3.2)	26.8 (3.1)	26.4 (3.0)	*Χ* ² = 2.41, *p* = .660
Displeasure in most activities	25.5 (3.0)	23.9 (3.0)	23.3 (2.9)	20.6 (2.8)	21.2 (2.8)	*Χ* ² = 1.91, *p* = .754
Inability to concentrate on task at hand	20.2 (2.8)	18.5 (2.7)	21.9 (2.9)	10.5 (2.1)	10.8 (2.1)	*Χ* ² = 17.55, *p* = .002
Depressed mood	16.8 (2.6)	18.5 (2.7)	17.1 (2.6)	12.0 (2.2)	10.4 (2.1)	*Χ* ² = 8.40, *p* = .078
Feeling irritable	11.5 (2.2)	12.7 (2.3)	12.9 (2.3)	8.6 (1.9)	7.1 (1.8)	*Χ* ² = 5.94, *p* = .204
Fatigue	15.4 (2.5)	13.6 (2.4)	10.5 (2.1)	7.2 (1.8)	5.2 (1.5)	*Χ* ² = 16.47, *p* = .002
Muscle tension	13.5 (2.4)	10.2 (2.1)	7.6 (1.8)	7.7 (1.9)	7.5 (1.8)	*Χ* ² = 6.64, *p* = .156

The mean-rank order of importance for indicators for the total sample, and by nation, are provided in Supplemental Table 3. Between-nation comparisons are reported in Table 4. Both wellbeing and mental health were represented in the top mean-ranked indicators of flourishing, with the highest mean-ranked indicator being the mental health item ‘*Experiencing pleasure in most activities*’. Two other mental health indicators ‘*Ability to concentrate on the task at hand*’ and ‘*Free of muscle tension*’ also had high mean-ranks. The top five mean-ranked items were rounded out by wellbeing indicators ‘*Feeling close to community and people in the local area*’ and ‘*Having a lot of energy*’. The only indicator for which a between-nation differences in mean-ranking was reported was for ‘*Having people around who really care about me*’ (*F* [4,367] = 4.79; *p* = .001), the overall sample’s top-ranked wellbeing item. Post-hoc analyses showed that Singaporean respondents had a higher mean-rank for this indicator compared to United Kingdom (*p* < .001) and Malaysian respondents (*p* = .006), but it is important to emphasise that despite these differences, it was highly endorsed across all nations compared to other indicators.

**Table 4.** Between nation differences in the rank scores of wellbeing and mental health indicators.

Wellbeing and Mental Health Indicators	Between nation differences
Having people around who really care about me	*F* = 4.79, *p* = .001
Feeling very positive about oneself	*F* = 1.03, *p* = .390
Feeling that what you do in your life is valuable and worthwhile	*F* = 2.39, *p* = .051
**A sense of worthlessness**	*F* = 2.40, *p* = .050
Being optimistic about the future	*F* = 0.76, *p* = .549
**Excessive worries and anxieties that are difficult to control**	*F* = 0.30, *p* = .879
Feeling calm and peaceful	*F* = 3.33, *p* = .011
**Poor quality sleep**	*F* = 2.94, *p* = .021
Taking all things together, generally feeling happy most of the time	*F* = 0.59, *p* = .671
**Depressed mood**	*F* = 1.25, *p* = .294
Having a sense of accomplishment	*F* = 0.58, *p* = .681
Being able to bounce back when things go wrong	*F* = 2.06, *p* = .086
**Fatigue**	*F* = 1.63, *p* = .172
**Feeling irritable**	*F* = 1.18, *p* = .324
Being interested in learning new things	*F* = 1.22; *p* = .301
Having a lot of energy	*F* = 0.42, *p* = .794
**Muscle tension**	*F* = 0.35, *p* = .846
**Inability to concentrate on task at hand**	*F* = 2.61, *p* = .038
Feeling close to community and people in the local area	*F* = 1.07, *p* = .374
**Displeasure in most activities**	*F* = 0.92, *p* = .452

*Note*. Mental health indicators in **bold** font. Items listed in order of highest to lowest rank score for the overall sample (see Supplemental Table 3). Mental health items were rephrased as the absence of pathology and preceded by ‘Not experiencing/Free of’.

**Figure 2.**
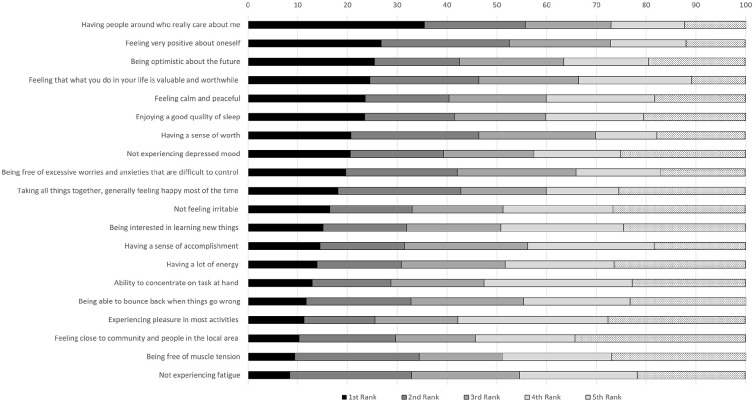
The proportion to which each item was ranked in top 5, total sample.

The proportion of all participants who endorsed each indicator within the top-5 is displayed in Figure 2 with stated prevalence reported for all nations provided in Supplemental Tables 4 to 8. There were very few between-nation differences (Table 5). Malaysian (*p* = .009) and South African respondents (*p* = .007) were less likely to rank the mental health indicator *‘Ability to Concentrate on the task at hand’* in the top 5 (*Χ*² (4) = 39.07, *p* = .007), and more likely to endorse the wellbeing indicator ‘*Feeling that what you do in your life is valuable and worthwhile*’ (*Χ*² (4) = 43.98; *p* =* .*002; South Africa: *p* = .021; Malaysia: *p* = .022), but the level of probability does not provide convincing evidence for a substantive or meaningful difference.

**Table 5.** Between nation differences in the proportion rank of wellbeing and mental health indicators.

Wellbeing and Mental Health Indicators	Between nation differences
Having people around who really care about me	*Χ* ² = 18.51; *p* = .295
Feeling very positive about oneself	*Χ* ² = 30.82; *p* = .014
Feeling that what you do in your life is valuable and worthwhile	*Χ* ² = 32.28; *p* = .009
**A sense of worthlessness**	*Χ* ² = 18.17; *p* = .314
Being optimistic about the future	*Χ* ² = 6.09; *p* = .987
**Excessive worries and anxieties that are difficult to control**	*Χ* ² = 17.24; *p* = .370
Feeling calm and peaceful	*Χ* ² = 25.37; *p* = .064
**Poor quality sleep**	*Χ* ² = 23.10; *p* = .111
Taking all things together, generally feeling happy most of the time	*Χ* ² = 13.63; *p* = .626
**Depressed mood**	*Χ* ² = 11.81; *p* = .757
Having a sense of accomplishment	*Χ* ² = 13.23; *p* = .656
Being able to bounce back when things go wrong	*Χ* ² = 17.60; *p* = .348
**Fatigue**	*Χ* ² = 13.57; *p* = .631
**Feeling irritable**	*Χ* ² = 17.04; *p* = .383
Being interested in learning new things	*Χ* ² = 13.21; *p* = .657
Having a lot of energy	*Χ* ² = 14.59; *p* = .555
**Muscle tension**	*Χ* ² = 16.03; *p* = .451
**Inability to concentrate on task at hand**	*Χ* ² = 23.49; *p* = .101
Feeling close to community and people in the local area	*Χ* ² = 18.51; *p* = .295
**Displeasure in most activities**	*Χ* ² = 11.37; *p* = .786

*Note*. Mental health indicators are in **bold** font. Items listed in order of highest to lowest rank score for the overall sample (see Supplemental Table 3). Mental health items were rephrased as the absence of pathology and preceded by ‘Not experiencing/Free of’.

### Socio-demographic and health characteristics that drive perceptions of flourishing

Bivariate associations for each wellbeing and mental health indicator with the covariates are reported in Table 6. As there were few substantive between-nation differences in the socio-demographic variables, and in the indicator endorsements and rankings, these analyses were undertaken on the total sample. There were no significant associations between items endorsed with sex, partner status or level of contact with mental illness. Age was positively associated with endorsing the wellbeing item ‘*Generally feeling happy most of the time*’ (*r* = .10, *p* < .001). Higher psychological distress and poorer wellbeing were associated with endorsing the mental health items ‘*Not experiencing fatigue*’ (K-10: *r* = .13, *p* < .001; WEMWBS *r* = −.15, *p* < .001), ‘*Being free of muscle tension*’ (K-10: *r* = .10, *p* < .001; WEMWBS: *r* = −.13, *p* < .001) and ‘*Being free of depressed mood*’ (K-10: *r* = .15, *p* < .001; WEMWBS: *r* = −.17, *p* < .001) as flourishing. Despite the high level of probability reported, the magnitude of these correlations is very small.

**Table 6.** Correlations between socio-demographic variables and endorsing indicators of mental health and wellbeing as important for flourishing.

Wellbeing and Mental Health Indicators	Age	Male	Partnered	Level of contact	K-10	WB
Wellbeing indicators						
Feeling close to community and people in the local area	−0.01	0.04	0.06	0.06	0.03	0.07
Having a lot of energy	−0.06	0.01	0.02	0.02	0.07	0.002
Having a sense of accomplishment	−0.04	0.05	0.02	−0.06	−0.05	0.06
Feeling very positive about oneself	0.01	0.07	−0.02	−0.01	−0.07	0.05
Interested in learning new things	−0.05	0.01	−0.03	−0.05	−0.02	0.04
Having people around who really care about me	−0.02	−0.03	−0.03	0.02	−0.03	−0.01
Being able to bounce back when things go wrong	0.04	−0.01	−0.03	−0.01	−0.04	0.01
Generally feeling happy most of the time	0.10*	−0.04	0.03	−0.06	−0.01	0.06
Being optimistic about the future	0.07	−0.05	0.02	−0.07	−0.10	0.07
Feeling what you do in life is valuable and worthwhile	0.03	−0.04	0.05	−0.02	−0.05	0.06
Feeling calm and peaceful	0.03	−0.06	0.03	0.01	0.01	−0.04
Mental health indicators						
A sense of worthlessness	0.03	0.05	−0.01	−0.01	−0.01	−0.01
Inability to concentrate on task at hand	−0.07	0.05	0.03	−0.04	0.04	0.06
Muscle tension	−0.08	0.03	0.01	0.05	0.10*	−0.13*
Poor quality sleep	0.03	−0.02	−0.03	0.05	0.02	−0.04
Fatigue	−0.04	0.01	0.01	0.08	0.13*	−0.15*
Displeasure in most activities	0.08	−0.01	−0.02	−0.02	−0.05	0.07
Feeling irritable	−0.06	0.07	−0.03	0.01	0.08	−0.07
Excessive worries and anxieties that are difficult to control	−0.01	−0.02	0.01	0.03	0.04	−0.07
Depressed mood	−0.06	−0.02	−0.05	0.05	0.15*	−0.17*

*Note*. Mental health items were rephrased as the absence of pathology and preceded by ‘Not experiencing/Free of’. K-10 = Kessler psychological distress scale; WB = Warwick Edinburgh wellbeing scale.

* *p* < .001.

We extended these correlations and further examined the relationship between these personal characteristics with the likelihood of endorsing each indicator as flourishing using a multi-variate logistic regression. After controlling for the socio-demographic and health characteristics, only the mental health indicator ‘*Not experiencing a depressed mood*’ was significantly associated with wellbeing (*OR* = 0.97; 95% CI [0.95, 0.98]; *p* < .001). At a less stringent level of probability (*p* < .010), wellbeing was also associated with endorsing wellbeing indicator ‘*Feeling close to community and people in the local area*’ (*OR* = 1.03; 95% CI [1.01, 1.04]; *p* = .004) and mental health indicator *‘Not experiencing fatigue’* (*OR* = 0.97; 95% CI [0.95, 0.99]; *p* = .007). However, these effects are of a very small magnitude and should not be over-emphasised as major predictors of the odds of endorsing the items.

## Discussion

The current study sought to extend Burns et al. (2022) by which community members endorsed and ranked wellbeing and mental health statements, drawn from a priori measures, that they thought best reflected flourishing. Our paper extends this initial study with a large age- and sex-balanced cross-national sample derived from five nations. Three main aims guided our analyses. Our first aim was to examine the extent to which participants would select a combination of wellbeing and mental health indicators as reflecting flourishing. Overall, whilst wellbeing indicators were slightly more endorsed than those indicators reflecting an absence of psychopathology, three mental health indicators ‘*sense of worth*’, ‘*quality of sleep*’ and ‘*free of excessive worries and anxieties*’ were ranked amongst the top-10 indicators. Notably, no single indicator was endorsed by a majority of respondents. These results correspond with those reported by Burns et al. (2022), and also Delle Fave et al. (2011) cross-national work in the related areas of happiness and meaning. The results of the ranking of individual indicators generally identified the more frequently endorsed items as reporting higher rank order. The endorsement and rank of individual indicators were generally consistent between nations; any differences were only of a small difference and on the least endorsed indicators. The results identified by Burns et al. (2022) appear to be consistent between nations and confirms our second aim.

For our third aim, we proposed that there would be no substantive associations between endorsement of individual indicators and respondents’ socio-demographic characteristics. There were several correlations of between *r* = .10 and *r* = .15 which were reported with high probability (*p* < .001), but the overall magnitude of these bivariate effects were small. In a multi-variate analysis by which endorsement of individual items were regressed on all the characteristics, only three very small effects were reported. Specifically, individuals reporting higher wellbeing were less likely to endorse *‘not experiencing a depressed mood’* (*OR* = 0.97; *p* < .001) and *‘not experiencing fatigue’* (*OR* = 0.97; *p* = .007) and more likely to endorse *‘feeling close to community and people in the local area’* (*OR* = 1.03; *p* = .004) as reflective of flourishing. These odds ratios are very small and we argue that our third hypothesis is also supported because the associations between socio-demographic factors and the odds of endorsing individual indicators were not of a substantive magnitude.

Despite the increasing utilization of flourishing as an important framework by which to describe individuals’ state of high wellbeing, it has been suggested that the rhetoric around flourishing is under-developed (Agenor et al., 2017). The failure to develop a unified and consistent flourishing framework can be related to substantive differences between frameworks which differentiate personal (Diener et al., 2010; Hone et al., 2014; Huppert & So, 2013; Seligman, 2018; VanderWeele, 2017), and broader societal policy-related (Corbin et al., 2021; Government, 2020; Sweden, 2017; Treasury, 2019) indicators. But even at the individual level, flourishing is frequently focused on wellbeing only. For example, Weęziak-Białowolska et al. (2019) compared the factor structure of the five-factor Flourishing Index (FI) and six-factor Secure Flourishing Index (SFI) between US financial institution workers, and clothing workers from Sri Lanka, Cambodia, China and Mexico. Similarly, Huppert and So (2013) compared flourishing, defined by the European Social Survey (ESS) wellbeing module (Huppert et al., 2009), across European nations within the ESS. And Santini et al. (2020) compared age and sex flourishing prevalence between Danish, Dutch and Canadian participants with flourishing derived from the Mental Health Continuum – Short Form scale (Keyes et al., 2008). But these between-nation analyses focus on levels of individual wellbeing indicators and overall flourishing from pre-defined flourishing model which ignore the role of psychopathology which were identified in this study, as well as in Burns et al. (2022).

Several cross-national studies have sought to identify individuals’ qualitative responses to related concepts such as happiness and wellbeing. In one qualitative study from the mid-west United States, 167 interviewees were asked to identify what people need to flourish (Willen, 2022). They reported social support (71%), stable income (70%) and social-determinants of health (69%); meaningful work, identity and family were also widely reported (44%–56%) by respondents. Delle Fave et al. (2011) reported that family and relationships were identified by between 27% and 29% of their multi-national samples as themes that reflected happiness, while other concepts like health, standard of living, work, religion and community were reported by 12% or less of respondents. When asked to describe the things most meaningful to them, 40% of respondents clearly separated themes of family from all other factors, including work, interpersonal relationships, health, personal growth (which were reported by <2 to 25.3% of the participants). Notably, not a single indicator was consistently identified to define happiness and meaning by the overwhelming majority of respondents. This points to the challenges wellbeing researchers and policy makers face in seeking to promote wellbeing as an important public policy outcome. Following those things that people prioritized as important for happiness and meaning, we propose flourishing will be similarly varied and individualistic, and support the notion that wellbeing, and consequently flourishing, are multidimensional and unlike current flourishing frameworks may comprise broader psychological health, encompassing both the presence of wellbeing and the absence of pathology.

Several limitations are worth discussion. First, we note that our cross-national sample reflects upper-middle- and high-income countries; it will be important to determine whether those from low and low-middle income countries respond in similar ways. In the current study, it may be possible to examine differences between respondents of varying levels of financial wellbeing between individuals within nations. We strongly recommend further consideration of this question with a broader range of nations. Related to the nations selected for this study, we would emphasise that we chose nations that differed in cultural norms, but otherwise were nations where English was either the official language, one of several official languages, or widely spoken. For example, Australia and the UK are predominantly English-speaking nations, with significant numbers of citizens for whom English is a second language, but nevertheless conversant in English. The other nations vary in the extent to which English is spoken. In Singapore and South Africa, English is one of several official languages. Specifically in Singapore, English is widely spoken; it is the main language of instruction in education, and is the common language in administration and government. And in South Africa 12 languages hold official language statues, but English is most widely spoken, and the overwhelming majority of education is undertaken in English, as is most business and government administration. In contrast, in Malaysia, Malay is the official language, but English dominates, trade, industry, education and is widely understood, particularly in city and urban areas. Finally, it should be noted that the national samples were drawn from Qualtrics. We recognise that the samples are not weighted to the population and that individuals who elect to join survey panels for reimbursement may be biased. However, previous analysis does suggest that data from Qualtrics panels is generally of good quality and samples are generally reflective of the population frames from which they are drawn (Armstrong et al., 2021; Belliveau et al., 2022; Boas et al., 2020; Ibarra et al., 2018; Peer et al., 2022). Fundamentally, further research is needed to consider confirming the findings from the present study, although we note that patterns of results are consistent with earlier findings (Burns et al., 2022).

Moving forward, there is clearly a need for more high quality qualitative research, of international and cross-cultural scope, to consider how individuals themselves define flourishing beyond those frameworks defined by wellbeing researchers themselves (Willen, 2022). Willen (2022) emphasized how flourishing has increasingly become an important area of discussion in psychology, public health, nursing, medicine and ethics, but that discussion in these areas is often divergent, and even cross-use of terminology where terms like flourishing, happiness and wellbeing might be used interchangeably, emphasizing Marsh et al. (2020) concerns about Jingle-Jangle wellbeing fallacies. And while VanderWeele et al. (2022) emphasized need for interdisciplinary research, highlighting their work with the Harvard Human Flourishing Program, multidisciplinary and transdisciplinary approaches (Choi & Pak, 2006) can also be championed as methods by which to further develop flourishing frameworks.

One proposition we have been contemplating however, is whether wellbeing researchers and policy advocates need yet *another* flourishing/wellbeing framework which purports to be the ‘gold-standard’. Currently, at the individual level, there are 3 or 4 ubiquitous models of flourishing that are typically cited in the literature, but as Hone et al. (2014) demonstrated, prevalence and concordance varies substantially between them. Based on the results of this study, which confirms with the work of Burns et al. (2022) with a similar methodology, and the early work of Delle Fave et al. (2011) into the related areas of happiness and meaning, it is clear that those things that community members prioritize is highly varied. Therefore, we question the development of yet another multi-dimensional model of flourishing that is reflective of the diversity of experiences/opinions. How many different components are needed in a framework before additional components contribute little additional information to some higher order flourishing/wellbeing construct?

In pondering these questions, we have begun to debate an alternative approach that may simplify how researchers and policy-makers may approach assessing the extent to which individuals are flourishing (and conversely languishing). As with common items that assess global health states (e.g. Self-Rated Health [SRH] or Life Satisfaction), perhaps an alternative method would simply be the utilization of a single item (or 2–3 item bank) that asks individuals the extent to which they feel/believe/assess their sense of flourishing. All the domains of wellbeing which currently constitute existing flourishing frameworks may well contribute and be associated with the individuals’ response to the single overall flourishing question, but rather than seeking a penultimate flourishing framework, which may or may not capture all of those elements of the lived experience that are valued by all, individual respondents determine for themselves (1) how to define ‘flourishing’ and (2) whether they judge/feel that they are flourishing. In the meantime, the flourishing literature is inhibited by (1) a lack of clear definition, (2) components that differ between frameworks, (3) community members prioritising a range of wellbeing indicators that may or may not be reflected in the framework they are presented and (4) a failure to consider concurrent pathology and the extent to which community members can flourish when experiencing ill-health.

To conclude, we have confirmed in this study that community members endorse a wide range of wellbeing indicators as reflecting flourishing, but they also endorse the absence of pathology, not just the presence of wellbeing. This suggests that flourishing frameworks need to consider the extent of the experience (or absence) of psychopathology is valued as highly as the experience (presence) of wellbeing. Notably, key socio-demographic characteristics, including exposure to prior mental illness, and personal levels of distress and wellbeing, were not substantively related to those items endorsed. These findings extend the current literature base by confirming a finding (Burns et al., 2022) to be consistent across five nations.

## Supplemental Material

sj-docx-2-isp-10.1177_00207640251323345 – Supplemental material for Cross-national variation in how members of the community define flourishing mental health Supplemental material, sj-docx-2-isp-10.1177_00207640251323345 for Cross-national variation in how members of the community define flourishing mental health by Richard Andrew Burns, Kerry Sargent and Dimity Ann Crisp in International Journal of Social Psychiatry

sj-tif-1-isp-10.1177_00207640251323345 – Supplemental material for Cross-national variation in how members of the community define flourishing mental health Supplemental material, sj-tif-1-isp-10.1177_00207640251323345 for Cross-national variation in how members of the community define flourishing mental health by Richard Andrew Burns, Kerry Sargent and Dimity Ann Crisp in International Journal of Social Psychiatry
