# Supplementary material for: Cross-national variation in how members of the community define flourishing mental health
Source: Int J Soc Psychiatry. 2025 Feb 28;71(6):1067–77. doi: 10.1177/00207640251323345 (PMC12357983; doi:10.1177/00207640251323345)
Supplement: sj-docx-2-isp-10.1177_00207640251323345 – Supplemental material for Cross-national variation in how members of the community define flourishing mental health [file sj-docx-2-isp-10.1177_00207640251323345.docx]

Supplementary Table 1*. Socio-demographic and health characteristics of the participants*

|  | Total |
| --- | --- |
|  | N=1044 |
| Male, N(%) | 523 (50.1%) |
| Partnered, N(%) | 739 (70.8%) |
| Age, M(SD) | 47.31 (16.28) |
| Level of Contact, M(SD) | 7.71 (3.92) |
| K-10, M(SD) | 21.42 (9.36) |
| Wellbeing, M(SD) | 47.26 (10.95) |

Supplementary Table 2. *Proportion of participants endorsing indicators as Flourishing*

|  | Total |
| --- | --- |
|  | % (SE) |
| Feeling calm and peaceful | 38.3 (1.5) |
| Feeling that what you do in your life is valuable and worthwhile | 36.2 (1.5) |
| Having people around who really care about me | 35.6 (1.5) |
| Feeling very positive about oneself | 35.4 (1.5) |
| Being optimistic about the future | 33.7 (1.5) |
| **A sense of worthlessness** | 33.0 (1.5) |
| Having a sense of accomplishment | 31.8 (1.4) |
| **Poor quality sleep** | 29.6 (1.4) |
| **Excessive worries & anxieties that are difficult to control** | 28.3 (1.4) |
| Taking all things together, generally feeling happy most of the time | 27.6 (1.4) |
| Being able to bounce back when things go wrong | 25.8 (1.4) |
| Being interested in learning new things | 24.2 (1.3) |
| **Displeasure in most activities** | 22.9 (1.3) |
| Having a lot of energy | 19.3 (1.2) |
| Feeling close to community and people in the local area | 16.9 (1.2) |
| **Inability to concentrate on task at hand** | 16.4 (1.1) |
| **Depressed mood** | 14.9 (1.1) |
| **Feeling irritable** | 10.5 (1.0) |
| **Fatigue** | 10.3 (0.9) |
| **Muscle tension** | 9.3 (0.9) |

Note, mental health items were rephrased as the absence of pathology and preceded by “Not experiencing/Free of”.

Supplementary Table 3. Mean rank scores for the wellbeing and mental health indicators for the overall sample and by Nation

|  | Total | Australia | UK | Singapore | South Africa | Malaysia |
| --- | --- | --- | --- | --- | --- | --- |
|  | Mean (SD) | Mean (SD) | Mean (SD) | Mean (SD) | Mean (SD) | Mean (SD) |
| Having people around who really care about me | 2.48 (1.42) | 2.53 (1.41) | 2.11 (1.28) | 3.08 (1.49) | 2.52 (1.37) | 2.29 (1.39) |
| Feeling very positive about oneself | 2.60 (1.34) | 2.41 (1.20) | 2.54 (1.31) | 2.79 (1.39) | 2.72 (1.39) | 2.47 (1.38) |
| Feeling that what you do in your life is valuable and worthwhile | 2.74 (1.34) | 2.77 (1.46) | 2.87 (1.40) | 2.39 (1.25) | 2.61 (1.25) | 3.00 (1.31) |
| **A sense of worthlessness** | 2.81 (1.38) | 2.67 (1.28) | 2.99 (1.43) | 2.82 (1.47) | 2.50 (1.34) | 3.15 (1.32) |
| Being optimistic about the future | 2.88 (1.46) | 2.80 (1.46) | 2.95 (1.42) | 2.72 (1.46) | 2.83 (1.46) | 3.11 (1.49) |
| **Excessive worries & anxieties that are difficult to control** | 2.89 (1.36) | 2.77 (1.45) | 3.02 (1.31) | 2.83 (1.30) | 2.96 (1.53) | 2.89 (1.25) |
| Feeling calm and peaceful | 2.94 (1.44) | 3.14 (1.38) | 3.4 (1.40) | 2.62 (1.44) | 2.90 (1.31) | 2.82 (1.52) |
| **Poor quality sleep** | 2.96 (1.46) | 3.24 (1.51) | 3.02 (1.55) | 2.46 (1.34) | 3.23 (1.41) | 2.88 (1.41) |
| Taking all things together, generally feeling happy most of the time | 3.05 (1.46) | 3.22 (1.48) | 3.02 (1.43) | 3.16 (1.47) | 2.88 (1.45) | 2.92 (1.50) |
| **Depressed mood** | 3.08 (1.48) | 3.03 (1.40) | 2.68 (1.60) | 3.14 (1.44) | 3.32 (1.41) | 3.48 (1.53) |
| Having a sense of accomplishment | 3.16 (1.31) | 3.22 (1.33) | 3.38 (1.37) | 3.07 (1.32) | 3.08 (1.37) | 3.14 (1.15) |
| Being able to bounce back when things go wrong | 3.23 (1.33) | 3.08 (1.34) | 3.38 (1.40) | 3.67 (1.27) | 3.12 (1.30) | 3.03 (1.30) |
| **Fatigue** | 3.25 (1.28) | 3.19 (1.31) | 3.15 (1.23) | 3.32 (1.39) | 2.86 (1.23) | 4.09 (0.94) |
| **Feeling irritable** | 3.26 (1.44) | 2.30 (1.63) | 2.92 (1.60) | 3.44 (1.09) | 3.55 (1.42) | 3.64 (1.34) |
| Being interested in learning new things | 3.27 (1.39) | 3.06 (1.37) | 3.21 (1.36) | 3.37 (1.47) | 3.60 (1.36) | 3.07 (1.39) |
| Having a lot of energy | 3.30 (1.39) | 3.21 (1.44) | 3.11 (1.35) | 3.40 (1.22) | 3.42 (1.55) | 3.41 (1.48) |
| **Muscle tension** | 3.32 (1.36) | 3.42 (1.52) | 3.30 (1.22) | 3.56 (1.26) | 3.06 (1.48) | 3.19 (1.28) |
| **Inability to concentrate on task at hand** | 3.34 (1.33) | 3.19 (1.41) | 3.23 (1.15) | 3.04 (1.44) | 3.86 (1.36) | 3.87 (0.97) |
| Feeling close to community and people in the local area | 3.48 (1.40) | 3.71 (1.22) | 3.14 (1.52) | 3.66 (1.37) | 3.63 (1.33) | 3.32 (1.51) |
| **Displeasure in most activities** | 3.49 (1.33) | 3.34 (1.36) | 3.43 (1.27) | 3.33 (1.43) | 3.74 (1.27) | 3.64 (1.33) |

Note: Mental health indicators are in **bold** font. Mental health items were rephrased as the absence of pathology and preceded by “Not experiencing/Free of”.

Supplementary Table 4. *Proportion of rank order for wellbeing and mental health indicators, Australia*

|  | Rank 1 | Rank 2 | Rank 3 | Rank 4 | Rank 5 |
| --- | --- | --- | --- | --- | --- |
|  | % (SE) | % (SE) | % (SE) | % (SE) | % (SE) |
| Feeling calm and peaceful | 16.3 (4.1) | 18.8 (4.4) | 8.3 (5.6) | 20.8 (8.2) | 25.0 (8.8) |
| Feeling that what you do in your life is valuable and worthwhile | 25.0 (5.4) | 26.6 (5.5) | 12.5 (4.1) | 18.8 (4.9) | 17.2 (4.7) |
| Having people around who really care about me | 35.3 (5.8) | 16.2 (4.5) | 19.1 (4.8) | 19.1 (4.8) | 10.3 (3.7) |
| Feeling very positive about oneself | 27.6 (5.9) | 27.6 (5.9) | 27.6 (5.9) | 10.3 (4.0) | 6.9 (3.3) |
| Being optimistic about the future | 27.0 (5.2) | 18.9 (4.6) | 18.9 (4.6) | 17.6 (4.4) | 17.6 (4.4) |
| **A sense of worthlessness** | 19.7 (4.6) | 31.6 (5.3) | 22.4 (4.8) | 14.5 (4.0) | 11.8 (3.7) |
| Having a sense of accomplishment | 12.1 (4.3) | 19.0 (5.1) | 25.9 (5.7) | 20.7 (5.3) | 22.4 (5.5) |
| **Poor quality sleep** | 21.0 (5.2) | 14.5 (4.5) | 9.7 (3.8) | 29.0 (5.8) | 25.8 (5.6) |
| **Excessive worries & anxieties that are difficult to control** | 29.0 (5.8) | 14.5 (4.5) | 22.6 (5.3) | 17.7 (4.9) | 16.1 (4.7) |
| Taking all things together, generally feeling happy most of the time | 17.5 (5.0) | 17.5 (5.0) | 17.5 (5.0) | 19.3 (5.2) | 28.1 (6.0) |
| Being able to bounce back when things go wrong | 10.0 (4.2) | 32.0 (6.6) | 20.0 (5.7) | 16.0 (5.2) | 22.0 (5.9) |
| Being interested in learning new things | 17.1 (6.3) | 17.1 (6.3) | 28.6 (7.6) | 17.1 (6.4) | 20.0 (6.8) |
| **Displeasure in most activities** | 11.3 (4.4) | 18.9 (5.4) | 17.0 (5.2) | 30.2 (6.3) | 22.6 (5.7) |
| Having a lot of energy | 16.3 (5.6) | 18.6 (5.9) | 18.6 (5.9) | 20.9 (6.2) | 25.6 (6.7) |
| Feeling close to community and people in the local area | 5.8 (4.0) | 8.8 (4.8) | 29.4 (7.8) | 20.6 (6.9) | 35.3 (8.2) |
| **Inability to concentrate on task at hand** | 19.0 (6.1) | 11.9 (5.0) | 21.4 (6.3) | 26.2 (6.8) | 21.4 (6.3) |
| **Depressed mood** | 17.1 (6.4) | 22.8 (7.1) | 20.0 (6.8) | 20.0 (6.8) | 20.0 (6.8) |
| **Feeling irritable** | 29.2 (9.3) | 16.7 (7.6) | 8.3 (5.6) | 20.8 (8.3) | 25.0 (8.8) |
| **Fatigue** | 9.4 (5.2) | 25.0 (7.6) | 25.0 (7.6) | 18.8 (6.9) | 21.9 (7.3) |
| **Muscle tension** | 17.8 (7.2) | 10.7 (5.8) | 17.9 (7.2) | 17.9 (7.2) | 35.7 (9.1) |

Note: Mental health indicators are in **bold** font. Mental health items were rephrased as the absence of pathology and preceded by “Not experiencing/Free of”.

Supplementary Table 5. *Proportion of rank order for wellbeing and mental health indicators, United Kingdom*

|  | Rank 1 | Rank 2 | Rank 3 | Rank 4 | Rank 5 |
| --- | --- | --- | --- | --- | --- |
|  | % (SE) | % (SE) | % (SE) | % (SE) | % (SE) |
| Feeling calm and peaceful | 14.3 (4.4) | 14.3 (4.4) | 15.9 (4.6) | 28.6 (5.7) | 27.0 (5.6) |
| Feeling that what you do in your life is valuable and worthwhile | 21.7 (5.0) | 24.6 (5.2) | 13.0 (4.1) | 26.1 (5.3) | 14.5 (4.2) |
| Having people around who really care about me | 44.6 (5.8) | 23.0 (4.9) | 17.6 (4.4) | 6.8 (2.9) | 8.1 (3.2) |
| Feeling very positive about oneself | 26.9 (5.0) | 26.9 (5.0) | 21.8 (4.7) | 14.1 (3.9) | 10.3 (3.4) |
| Being optimistic about the future | 20.3 (5.2) | 20.3 (5.2) | 23.7 (5.6) | 15.3 (4.7) | 20.3 (5.2) |
| **A sense of worthlessness** | 19.7 (4.7) | 21.1 (4.8) | 21.1 (4.8) | 16.9 (4.4) | 21.1 (4.8) |
| Having a sense of accomplishment | 14.2 (4.7) | 12.5 (4.4) | 19.6 (5.3) | 28.6 (6.0) | 25.0 (5.7) |
| **Poor quality sleep** | 27.4 (5.7) | 11.3 (0.4) | 17.7 (4.9) | 19.4 (5.0) | 8.1 (3.2) |
| **Excessive worries & anxieties that are difficult to control** | 14.8 (4.8) | 20.4 (5.5) | 31.5 (6.3) | 14.8 (4.8) | 18.5 (5.3) |
| Taking all things together, generally feeling happy most of the time | 20.4 (5.5) | 16.7 (5.1) | 25.9 (5.9) | 14.8 (4.8) | 22.2 (5.7) |
| Being able to bounce back when things go wrong | 12.0 (4.6) | 18.0 (5.4) | 20.0 (5.7) | 20.0 (5.7) | 30.0 (6.4) |
| Being interested in learning new things | 13.5 (4.7) | 21.2 (5.7) | 17.3 (5.2) | 26.9 (6.2) | 21.2 (5.7) |
| **Displeasure in most activities** | 8.2 (3.9) | 20.4 (5.8) | 14.3 (5.0) | 34.7 (6.8) | 22.4 (5.9) |
| Having a lot of energy | 15.6 (5.4) | 15.6 (5.4) | 33.3 (7.1) | 13.3 (5.1) | 22.2 (6.2) |
| Feeling close to community and people in the local area | 17.1 (6.3) | 25.7 (7.4) | 11.4 (5.4) | 17.1 (6.4) | 28.6 (7.6) |
| **Inability to concentrate on task at hand** | 7.8 (4.4) | 21.1 (6.6) | 21.1 (6.6) | 39.5 (7.9) | 10.5 (5.0) |
| **Depressed mood** | 36.8 (7.8) | 13.2 (5.5) | 15.8 (5.9) | 13.2 (5.5) | 21.1 (6.6) |
| **Feeling irritable** | 26.9 (8.7) | 19.2 (7.7) | 15.4 (7.1) | 11.5 (6.3) | 26.9 (8.7) |
| **Fatigue** | 7.4 (5.0) | 29.6 (8.8) | 18.5 (7.5) | 29.6 (8.8) | 14.8 (6.9) |
| **Muscle tension** | 0 | 35.0 (10.7) | 25.0 (9.7) | 15.0 (7.9) | 25.0 (9.7) |

Note: Mental health indicators are in **bold** font. Mental health items were rephrased as the absence of pathology and preceded by “Not experiencing/Free of”.

Supplementary Table 6. Proportion of rank order for wellbeing and mental health indicators, Singapore

|  | Rank 1 | Rank 2 | Rank 3 | Rank 4 | Rank 5 |
| --- | --- | --- | --- | --- | --- |
|  | % (SE) | % (SE) | % (SE) | % (SE) | % (SE) |
| Feeling calm and peaceful | 31.4 (5.0) | 19.8 (4.3) | 19.8 (4.3) | 14.0 (3.7) | 15.1 (3.9) |
| Feeling that what you do in your life is valuable and worthwhile | 31.3 (5.7) | 26.9 (5.4) | 19.4 (4.8) | 16.4 (4.5) | 6.0 (2.9) |
| Having people around who really care about me | 18.5 (4.8) | 26.2 (5.5) | 9.2 (3.6) | 21.5 (5.1) | 24.6 (5.3) |
| Feeling very positive about oneself | 22.1 (5.0) | 23.5 (5.1) | 25.0 (5.3) | 11.8 (3.9) | 17.6 (4.6) |
| Being optimistic about the future | 29.7 (5.7) | 17.2 (4.7) | 21.9 (5.2) | 14.1 (4.3) | 17.2 (4.7) |
| **A sense of worthlessness** | 24.6 (5.7) | 21.1 (5.4) | 22.8 (5.6) | 10.5 (4.1) | 21.1 (5.4) |
| Having a sense of accomplishment | 18.3 (4.3) | 13.4 (3.8) | 25.6 (4.8) | 28.0 (5.0) | 14.6 (3.9) |
| **Poor quality sleep** | 31.1 (5.9) | 26.2 (5.6) | 18.0 (4.9) | 14.8 (4.5) | 9.8 (3.8) |
| **Excessive worries & anxieties that are difficult to control** | 18.2 (4.7) | 24.2 (5.3) | 27.2 (5.5) | 16.7 (4.6) | 13.6 (4.2) |
| Taking all things together, generally feeling happy most of the time | 15.6 (4.5) | 25.0 (5.4) | 15.6 (4.5) | 15.6 (4.5) | 28.2 (5.6) |
| Being able to bounce back when things go wrong | 8.7 (4.2) | 10.9 (4.6) | 15.2 (5.3) | 34.8 (7.0) | 30.4 (6.8) |
| Being interested in learning new things | 18.4 (5.5) | 12.2 (4.7) | 10.2 (4.3) | 32.7 (6.7) | 26.5 (6.3) |
| **Displeasure in most activities** | 18.4 (5.5) | 10.2 (4.3) | 16.3 (5.3) | 30.6 (6.6) | 24.5 (6.1) |
| Having a lot of energy | 6.0 (3.4) | 22.0 (5.9) | 20.0 (5.7) | 30.0 (6.5) | 22.0 (5.9) |
| Feeling close to community and people in the local area | 6.9 (4.7) | 17.2 (7.0) | 20.7 (7.5) | 13.8 (6.4) | 41.4 (9.1) |
| **Inability to concentrate on task at hand** | 19.6 (5.8) | 19.6 (5.8) | 19.6 (5.8) | 19.6 (5.8) | 21.7 (6.1) |
| **Depressed mood** | 16.7 (6.2) | 19.4 (6.6) | 22.2 (6.9) | 16.7 (6.2) | 25.0 (7.2) |
| **Feeling irritable** | 3.7 (3.6) | 14.8 (6.8) | 33.3 (9.1) | 29.6 (8.8) | 18.5 (7.5) |
| **Fatigue** | 13.6 (7.3) | 13.6 (7.3) | 27.3 (9.5) | 18.2 (8.2) | 27.3 (9.5) |
| **Muscle tension** | 0 | 31.3 (11.6) | 12.5 (8.3) | 25.0 (10.8) | 31.3 (11.6) |

Note: Mental health indicators are in **bold** font. Mental health items were rephrased as the absence of pathology and preceded by “Not experiencing/Free of”.

Supplementary Table 7. Proportion of rank order for wellbeing and mental health indicators, South Africa

|  | Rank 1 | Rank 2 | Rank 3 | Rank 4 | Rank 5 |
| --- | --- | --- | --- | --- | --- |
|  | % (SE) | % (SE) | % (SE) | % (SE) | % (SE) |
| Feeling calm and peaceful | 18.1 (4.5) | 22.2 (4.9) | 25.0 (5.1) | 20.8 (4.8) | 13.9 (4.1) |
| Feeling that what you do in your life is valuable and worthwhile | 28.7 (4.9) | 12.6 (3.6) | 33.3 (5.1) | 19.5 (4.3) | 5.7 (2.5) |
| Having people around who really care about me | 32.9 (5.4) | 18.4 (4.4) | 22.4 (4.8) | 15.7 (4.2) | 10.5 (3.5) |
| Feeling very positive about oneself | 25.2 (4.7) | 24.1 (4.6) | 16.1 (3.9) | 21.8 (4.4) | 12.6 (3.6) |
| Being optimistic about the future | 27.2 (4.9) | 17.3 (4.2) | 18.5 (4.3) | 19.8 (4.4) | 17.3 (4.2) |
| **A sense of worthlessness** | 26.6 (4.9) | 30.4 (5.2) | 24.1 (4.8) | 3.8 (2.2) | 15.2 (4.1) |
| Having a sense of accomplishment | 15.8 (4.2) | 22.4 (4.8) | 19.7 (4.6) | 22.4 (4.8) | 19.7 (4.6) |
| **Poor quality sleep** | 17.0 (5.2) | 13.2 (4.7) | 24.5 (5.9) | 20.8 (5.6) | 24.5 (5.9) |
| **Excessive worries & anxieties that are difficult to control** | 23.2 (5.6) | 23.2 (5.6) | 10.7 (4.1) | 19.6 (5.3) | 23.2 (5.6) |
| Taking all things together, generally feeling happy most of the time | 17.6 (5.3) | 35.3 (6.7) | 9.8 (4.2) | 15.7 (5.1) | 21.6 (5.8) |
| Being able to bounce back when things go wrong | 13.6 (4.5) | 18.6 (5.1) | 28.8 (5.9) | 20.3 (5.2) | 18.6 (5.1) |
| Being interested in learning new things | 10.2 (3.9) | 13.6 (4.5) | 20.3 (5.2) | 22.0 (5.4) | 33.9 (6.2) |
| **Displeasure in most activities** | 9.3 (4.4) | 7.0 (3.9) | 18.6 (5.9) | 30.2 (7.0) | 17.6 (5.3) |
| Having a lot of energy | 19.2 (7.7) | 11.5 (6.3) | 11.5 (6.3) | 23.1 (8.3) | 34.6 (9.3) |
| Feeling close to community and people in the local area | 7.5 (4.2) | 20 (6.3) | 7.5 (4.2) | 32.5 (7.4) | 32.5 (7.4) |
| **Inability to concentrate on task at hand** | 9.1 (6.1) | 9.1 (6.1) | 13.6 (7.3) | 22.7 (8.9) | 45.5 (10.6) |
| **Depressed mood** | 12.0 (6.4) | 20.0 (8.0) | 20.0 (8.0) | 20.0 (8.0) | 28.0 (9.0) |
| **Feeling irritable** | 11.1 (7.4) | 16.7 (8.8) | 11.1 (7.4) | 27.8 (10.6) | 33.3 (11.1) |
| **Fatigue** | 7.1 (6.9) | 42.9 (13.2) | 21.4 (11.0) | 14.3 (9.4) | 14.3 (9.4) |
| **Muscle tension** | 18.8 (9.8) | 25.0 (10.8) | 6.3 (6.1) | 31.3 (11.6) | 18.8 (9.8) |

Note: Mental health indicators are in **bold** font. Mental health items were rephrased as the absence of pathology and preceded by “Not experiencing/Free of”.

Supplementary Table 8. Proportion of rank order for wellbeing and mental health indicators, Malaysia

|  | Rank 1 | Rank 2 | Rank 3 | Rank 4 | Rank 5 |
| --- | --- | --- | --- | --- | --- |
|  | % (SE) | % (SE) | % (SE) | % (SE) | % (SE) |
| Feeling calm and peaceful | 32.7 (4.7) | 10.2 (3.1) | 17.3 (3.8) | 22.4 (4.2) | 17.3 (3.8) |
| Feeling that what you do in your life is valuable and worthwhile | 17.0 (4.0) | 21.6 (4.4) | 18.2 (4.1) | 30.7 (4.9) | 12.5 (3.5) |
| Having people around who really care about me | 42.7 (5.2) | 18.0 (4.1) | 16.8 (4.0) | 12.4 (3.5) | 10.1 (3.2) |
| Feeling very positive about oneself | 32.1 (5.3) | 26.9 (5.0) | 14.1 (3.9) | 15.4 (4.1) | 11.5 (3.6) |
| Being optimistic about the future | 22.2 (4.9) | 12.5 (3.9) | 22.2 (4.9) | 18.1 (4.5) | 25.0 (5.1) |
| **A sense of worthlessness** | 11.9 (4.2) | 22.0 (5.4) | 27.1 (5.8) | 16.9 (4.9) | 22.0 (5.4) |
| Having a sense of accomplishment | 10.2 (3.9) | 16.9 (4.9) | 33.9 (6.2) | 27.1 (5.8) | 11.9 (4.2) |
| **Poor quality sleep** | 20.6 (4.9) | 23.5 (5.1) | 22.1 (5.0) | 14.7 (4.3) | 19.1 (4.8) |
| **Excessive worries & anxieties that are difficult to control** | 12.5 (4.4) | 30.4 (6.1) | 26.7 (5.9) | 16.1 (4.9) | 14.3 (4.7) |
| Taking all things together, generally feeling happy most of the time | 19.7 (5.1) | 29.5 (5.8) | 16.4 (4.7) | 8.2 (3.5) | 26.2 (5.6) |
| Being able to bounce back when things go wrong | 13.1 (4.3) | 24.6 (5.5) | 26.2 (5.6) | 18.0 (4.9) | 18.0 (4.9) |
| Being interested in learning new things | 17.5 (5.0) | 19.3 (5.2) | 21.1 (5.4) | 22.8 (5.6) | 19.3 (5.2) |
| **Displeasure in most activities** | 8.9 (4.2) | 13.3 (5.1) | 17.8 (5.7) | 24.4 (6.4) | 35.6 (7.1) |
| Having a lot of energy | 16.2 (6.1) | 13.5 (5.6) | 16.2 (6.1) | 21.6 (6.8) | 32.4 (7.7) |
| Feeling close to community and people in the local area | 13.5 (5.6) | 24.3 (7.1) | 13.5 (5.6) | 13.5 (5.6) | 35.1 (7.8) |
| **Inability to concentrate on task at hand** | 0 | 13.0 (7.0) | 13.0 (7.0) | 47.8 (10.4) | 28.1 (9.2) |
| **Depressed mood** | 14.3 (7.6) | 19.0 (8.6) | 9.5 (6.4) | 19.0 (8.6) | 38.1 (10.6) |
| **Feeling irritable** | 7.1 (6.9) | 14.3 (9.3) | 21.4 (11.0) | 21.4 (11.0) | 35.7 (12.8) |
| **Fatigue** | 0 | 9.1 (8.7) | 9.1 (8.7) | 45.5 (15.0) | 36.4 (14.5) |
| **Muscle tension** | 6.3 (6.1) | 31.3 (11.6) | 18.7 (9.8) | 25.0 (10.8) | 18.8 (9.8) |

Note: Mental health indicators are in **bold** font. Mental health items were rephrased as the absence of pathology and preceded by “Not experiencing/Free of”.
