# Supplementary figures and images for: Cross-national variation in how members of the community define flourishing mental health
Source: Int J Soc Psychiatry. 2025 Feb 28;71(6):1067–77. doi: 10.1177/00207640251323345 (PMC12357983; doi:10.1177/00207640251323345)

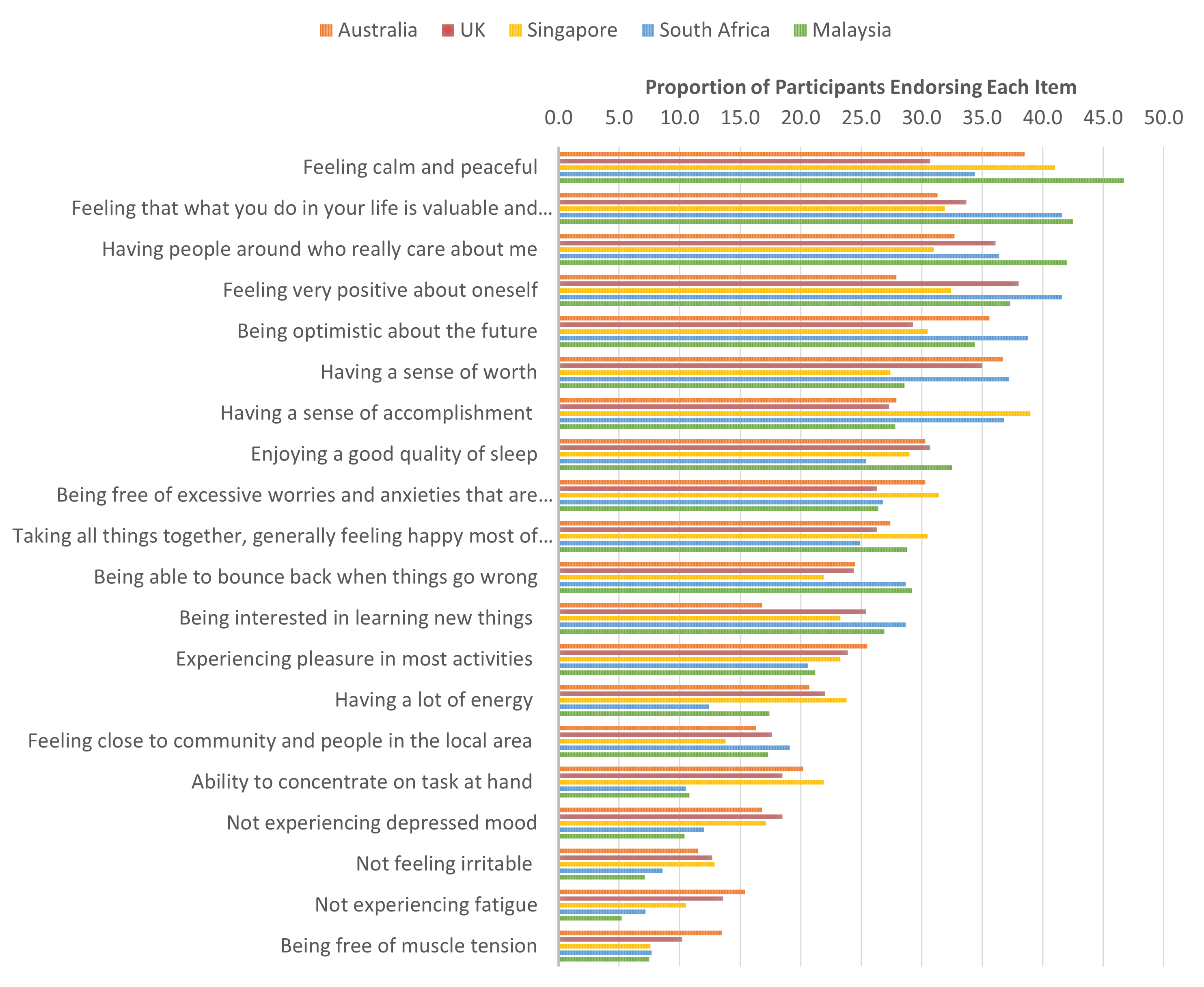

Supplement: sj-tif-1-isp-10.1177_00207640251323345 – Supplemental material for Cross-national variation in how members of the community define flourishing mental health [file sj-tif-1-isp-10.1177_00207640251323345.tif]
